# Recent Advances in Inflammation and Treatment of Small Airways in Asthma

**DOI:** 10.3390/ijms20112617

**Published:** 2019-05-28

**Authors:** Elisabetta Zinellu, Barbara Piras, Giulia G. M. Ruzittu, Sara S. Fois, Alessandro G. Fois, Pietro Pirina

**Affiliations:** 1 Respiratory Unit, Azienda Ospedaliero Universitaria (AOU), V.le San Pietro, 07100 Sassari, Italy; elisabetta.zinellu@aousassari.it; 2 Respiratory Unit, Department of Medical, Surgical and Experimental Sciences, University of Sassari, V.le San Pietro, 07100 Sassari, Italy; barbara.piras@aousassari.it (B.P.); giuliaruzittu@hotmail.com (G.G.M.R.); sara.solveig.fois@gmail.com (S.S.F.); agfois@uniss.it (A.G.F.)

**Keywords:** asthma, small airways, inflammation, treatment efficacy

## Abstract

Small airways were historically considered to be almost irrelevant in the development and control of pulmonary chronic diseases but, as a matter of fact, in the past few years we have learned that they are not so “silent”. Asthma is still a worldwide health issue due to the great share of patients being far from optimal management. Several studies have shown that the deeper lung inflammation plays a critical role in asthma pathogenesis, mostly in these not well-controlled subjects. Therefore, assessing the degree of small airways inflammation and impairment appears to be a pivotal step in the asthmatic patient’s management. It is now possible to evaluate them through direct and indirect measurements, even if some obstacles still affect their clinical application. The success of any treatment obviously depends on several factors but reaching the deeper lung has become a priority and, for inhaled drugs, this is strictly connected to the molecule’s size. The aim of the present review is to summarize the recent evidence concerning the small airway involvement in asthma, its physiopathological characteristics and how it can be evaluated in order to undertake a personalized pharmacological treatment and achieve a better disease control.

## 1. Introduction

Asthma is a common, chronic respiratory disease affecting 1–18% of the whole population in different countries and, despite the advances in diagnosis and treatment, it remains a serious global health problem with a great proportion of patients that are still not properly controlled [1].

The lack of asthma control is rarely associated to a real drug-resistant condition, but it is related to different factors regarding the patients (age, comorbidities, and cognitive status), their therapy compliance, the disease phenotypes, the drugs, and their formulations [2,3,4,5,6,7,8,9]. In particular, recent studies have indicated the inflammation in the small airways as a substantial feature associated with bronchial hyper-responsiveness, worsening of asthma symptoms, and increased exacerbations [10,11].

In this review, we focus our interest in the role played by the small airways and their inflammation in the physiopathological and clinical aspects of asthma and in the recent advances in treatment of this airway compartment. We performed a literature review via Pubmed database using the following terms: “asthma”, “small airways”, “inflammation”, “biomarkers”, “treatment”, “extrafine formulations” in different combination. We selected the studies performed on asthmatic patients on the basis of their relevance.

## 2. Small Airways Pathology

The lungs are segmentally divided from the trachea (generation 1) down to the alveoli (generation 23) [12]. The small airways are defined as airways with an internal diameter of less than 2 mm located from the eighth generation of airways to the respiratory bronchioles [13]. In the past, the small airways were considered as “silent” [14] because of their relatively low impact on the whole resistance of the respiratory tree in comparison with the larger airways in healthy subjects as demonstrated by Hoggs and others [15,16,17]. In 1992 Yanai et al. measured the intrabronchial pressure in living humans using a catheter-tapped micromanometer. By doing so, they could demonstrate how, in subjects with asthma and Chronic Obstructive Pulmonary Disease (COPD), peripheral airways are the main site of development of airflow obstruction [18]. The advent of new techniques such as morphometric and immunocytochemical techniques has allowed us to reveal how the inflammation affects the whole respiratory tree. The inflammatory response is driven by Th2 cells that migrate to the airway epithelium and to the subepithelial mucosa where they secrete cytokines that are responsible for mucus production, IgE synthesis, bronchial remodeling and for the recruitment of mast cells, basophils, and eosinophils. The inflammatory process produces a structural change leading to a thickening of the airway wall and to an increase of the interstitial matrix, with a consequent reduction of the airway lumen. High-resolution computed tomography (HRCT) imaging has revealed that the distal airways are a major site of airway obstruction in patients with asthma, while functional bronchoscopic studies have shown a high peripheral airflow resistance in asthmatic subjects [19]. Moreover, as described below, it has been shown that the inflammatory cells are more abundant in the distal portion of the airways.

Then, the inflammation in the distal lung has a crucial role in the development and the control of a disease with multiple clinical phenotypes [1].

## 3. Small Airways Inflammation and Assessment

Evaluating the functional and inflammatory impairment of peripheral airways could help in identifying the clinical phenotype and guide the treatment choices. Therefore, it should be a routine process in the evaluation of asthmatic subjects independently of disease control. Nowadays there are several direct and indirect measurements of peripheral airways involvement (Table 1), even if some of them are only performed for research purposes and are not ready yet for clinical routine use.

### 3.1. Inflammation and Biomarkers

By using invasive techniques such as endobronchial and transbronchial biopsies, or lung surgery, a number of investigators assessed the type of inflammatory cells, in the large and in the small airways of asthmatics. In these studies, the authors found an infiltrate rich of lymphocytes, eosinophils and macrophages in the distal lung compartment of asthmatic patients compared with the large airways and with control subjects [20,21,22,23]. Moreover, Minshall et al. showed increased IL-5 and IL-4 mRNA expression in the distal airways of asthmatic patients compared with non-asthmatic control subjects, and an increased expression of IL-5 mRNA expression in the distal airways compared with proximal airways [24]. Some authors have investigated if small airways inflammation could indicate the presence of a particular asthmatic phenotype. Kraft et al. have shown that patients with nocturnal symptoms have a greater inflammatory involvement of the small airways. In particular, they observed increased eosinophil and macrophage counts in the distal airways biopsies taken during the night compared with those taken in the afternoon. Moreover, they found out that patients with nocturnal symptoms had more eosinophil counts in the small airways against to proximal airways [25]. However, although these procedures have been useful to understand the contribution of the small airways in the pathogenesis of asthma, their invasiveness makes them not applicable in clinical practice.

A non-invasive measure of airways inflammation is represented by fractional exhaled nitric oxide concentration (FENO) in the exhaled air [26,27]. In allergic airway inflammation, the production of FENO is increased due to pro-inflammatory cytokines-mediated upregulation of inducible nitric oxide synthase (iNOS) [28,29]. The measurement of this biomarker at multiple exhalation flows allows to separate bronchial from peripheral/alveolar nitric oxide and, consequently, to discriminate between the inflammation coming from the larger and from the smaller airways [30,31,32]. In this context, alveolar nitric oxide has been largely studied and has been associated with the presence of symptoms and lack of asthma control. In particular, Mahut et al. found higher levels of alveolar NO in symptomatic children with asthma compared with asymptomatic asthmatic children [33]. Paraskakis et al. showed that alveolar nitric oxide was increased in asthmatic children compared to normal children. Moreover, the authors observed that children with poorly controlled asthma had higher alveolar nitric oxide levels in contrast with children with good asthma control [34]. Lehtimaki et al. demonstrated increased alveolar NO concentration in patients with asthmatic symptoms but normal lung function compared with control subjects [35]. The same authors, in a previous study, had found that asthmatic patients with nocturnal symptoms had higher alveolar NO levels against asthmatic patients with daily symptoms only [36]. Other authors confirmed the relationship between alveolar NO levels and lack of asthma control. In particular, Puckett et al. [37] investigated nitric oxide in asthmatic children dividing it into categories: normal bronchial and alveolar NO; elevated bronchial and normal alveolar NO; normal bronchial and elevated alveolar NO; elevated bronchial and alveolar NO. All the categories had similar lung function. Only the categories with increased alveolar NO were related to poor asthma control, as assessed by the asthma control test, and had severe exacerbations more frequently. In another study, Scichilone et al. found a significant correlation between the worsening of disease control assessed by the asthma control test and increasing alveolar nitric oxide in subjects with mild asthma. In addition, they observed that alveolar nitric oxide was significantly higher in patients with uncontrolled symptoms compared with those presenting well-controlled asthma [38]. Some studies have shown that a more intense degree of peripheral inflammation occurs in the most severe form of asthma. Brindicci et al. demonstrated higher levels of alveolar NO in patients with mild asthma respect to controls and also in severe asthma compared to mild asthma [39]. Higher levels of alveolar nitric oxide were also observed in patients with refractory asthma compared with patients with mild to moderate asthma [40]. Moreover, in this study, a strong positive correlation was shown between alveolar NO and BAL eosinophil cell counts. Van Veen et al. investigated the relationship between peripheral airway inflammation and dysfunction with asthma severity, finding a positive strong relationship between alveolar nitric oxide and parameters of peripheral airway dysfunction in patients with severe asthma [41]. An association between exhaled NO and small airway functions, as assessed by the single breath nitrogen test, was also found in mild atopic asthma [42]. In this study the small airways function was also correlated to exhaled 8-isoprostane, a biomarker of oxidative stress, produced by the reactive oxygen species-mediated peroxidation of arachidonic acid.

Another exhaled molecule that has recently been evaluated as biomarker of small airway inflammation in asthma is Leukotriene B4 (LTB4), a pro-inflammatory mediator whose concentration is elevated in inflammatory diseases [43]. Evaluating children with asthma and control subjects, it was found that the first group had significantly higher LTB4 levels in the small airways, while no difference was observed in the large airways [44].

Induced sputum has also been studied for the assessment of biomarkers of airways inflammation in asthma. Patients with chronic asthma appeared to have higher sputum levels of eosinophils (cells/ml and percent) and of Eosinophil Cationic Protein and lower levels of neutrophils (cells/ml and percent) and Neutrophilic elastase than patients with COPD [45]. Sputum induction is a non-invasive method that has been used to explore inflammatory cells especially in central airways, even if a procedure has been proposed in order to provide information on distal airways inflammation by fractional analysis of sequential induced sputum samples. In particular, it has been suggested that the duration of sputum induction could influence the measurement: induced sputum collected earlier originates from larger airways, whereas sputum collected later originates from smaller airways [46]. However, case-control studies that employ this method to investigate biomarkers of small airway inflammation in asthma are lacking.

Carboxymethyl-lysine, a glycoxidation product derived from sequential glycation and oxidation reactions between reducing sugars and proteins, has been studied as biomarker of oxidative damage to proteins. In particular, increased levels of this molecule were observed in induced sputum of asthmatic patients in a case-control study. Furthermore, it was inversely correlated with forced expiratory flow between 25% and 75% of forced vital capacity (FEF25–75), an established index of small airways obstruction [47]. Surfactant-protein D (SP-D), that is a molecule of the innate immune system involved in pulmonary host defense and modulation of allergic responses, is another biomarker recently correlated with index of peripheral airway dysfunction. Salivary SP-D levels were higher in asthmatic children than in healthy controls. Additionally, it correlated with parameters of peripheral airways resistance and with the severity of asthma exacerbation [48]. Surfactant protein A and albumin, measured as particles in exhaled Air (PExA), were also described to be associated with small airways dysfunction in asthmatic subjects [49].

Among the inflammatory markers that have been discussed, alveolar nitric oxide is the most studied and it seems to be more useful in clinical practice.

### 3.2. Functional Assessments

#### 3.2.1. Spirometry and Plethysmography

Proximal airways contribute mostly to measurements assessed during the early phase of expiration, including forced expiratory volume in 1 second (FEV_1_), whereas distal airways are believed to contribute most to the end of expiration [50]. Therefore, FEF_25–75%_ and FEF_50%_ could reflect distal airway obstruction, but their values are highly variable and can be used only if forced vital capacity (FVC) is normal [51,52].

During plethysmography, measures of expiratory air trapping such as the FVC and the ratio of residual volume (RV) to total lung capacity (TLC) are often assumed to represent small airway obstruction, even if there is no conclusive evidence about it [53]. Mechanisms leading to lung hyperinflation in asthmatics include expiratory airflow limitation and premature closure of small airways [54], activity of inspiratory muscles at the end of expiration and reduced pulmonary elasticity [55].

In the Severe Asthma research program, the FVC% showed an inverse correlation to the ratio of RV/TLC% [56] and Papi et al. demonstrated that improvement in FVC may reflect reduction in air trapping and small airway obstruction after a 3 months treatment with small particles (~1.5 microns) combination of inhaled corticosteroids (ICS) with long-acting B2-adrenoceptor agonists (LABA) [57].

#### 3.2.2. Pulmonary Resistance Measurements

Impulse oscillometry (IOS), a variant of the forced oscillation technique (FOT), uses the indices of reactance (X), resistance (R) and impedance (Z) to determine the airway function from different lung regions in response to a regular square wave of pressure 5 times per second. Lower frequencies, in particular modification of the frequency dependent resistance between 5 and 20 Hz (R5–R20 Hz) and capacitive reactance at 5 Hz (X5 Hz) reflect changes arising from more distal airways, whereas higher frequencies reflect changes from more central larger airways.

IOS was used in persistent asthmatic patients treated in primary care and Anderson and colleagues noted that R5–R20 was abnormal in about two-third of the patients at each step of the treatment guidelines. After treatment with small particle corticosteroid, the total resistance (R5) significantly lowered in comparison with the standard treatment even if there were not any differences in FEV_1_ between the two groups [58].

#### 3.2.3. Nitrogen Washout Tests

Ventilation heterogeneity of the small airways can be investigated with a nitrogen washout test. It can also differentiate the involvement of the small airways.

During the single-breath nitrogen washout test (SBWT) the subject, after an expiration to residual volume, slowly inhales a 100% concentration of oxygen to TLC. Then, he has to expire to residual volume again with the registration of the nitrogen tracing. This technique allows one to evaluate the peripheral ventilation inhomogeneity (slope of phase III) and airway closure (closing capacity). The index of closing volume (CV) reflects air-trapping due to early small airways closure and it has been shown to have greater sensitivity to small airways inflammation than traditional spirometric measures of forced expiratory flow between 25 to 75 percent of the forced vital capacity (FEF_25–75_) [59].

The multiple breath washout test (MBW) seems to have several advantages with respect to SBWT. In particular, it can distinguish between the different contribution from the proximal conducting airways compartments and the distal acinar regions through the S_cond_ and S_acin_ indices, derived from phase III slopes [60]. Their value increases when ventilation heterogeneity increases. In particular, S_acin_ value will increase if ventilation heterogeneity is increased in the acinar lung zone, while S_cond_ will increase if ventilation heterogeneity is increased in the conductive lung zone.

### 3.3. Imaging

In the past few years, imaging techniques have become an important instrument to evaluate the small airways thanks to their worldwide availability and homogeneous assessment criteria [61].

**Figure 1:**
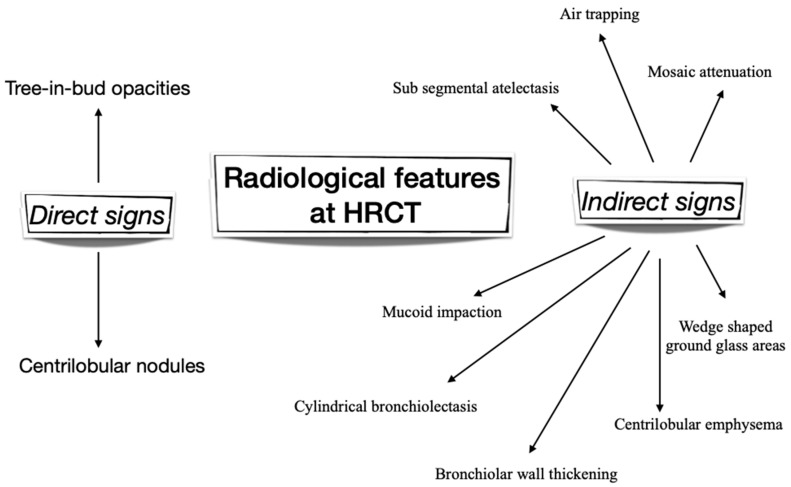
Radiological features of small airways disease at High Resolution Computed Tomography (HRCT).

Although the HRCT scanners resolution is higher than the small airways diameter, it is possible to evaluate small airways alteration (Figure 1). Small airways disease causes primarily air trapping that may be seen as areas of low attenuation distal to the site of obstruction even in the asthmatic subjects [62]. The comparison between the mean lung density between expiratory and inspiratory HRCT provides a quantitative measure called MLD_E/I_. In asthma, MLD_E/I_ correlates strongly with FEV_1_, FEV_1_/FVC ratio, FEF_25–75_, and RV/TLC, suggesting that it reflects small airways disease [63]. Air trapping is more marked during acute exacerbations of asthma and shows responsiveness to steroids [64]. It is correlated to hospitalization, to a worse pulmonary obstruction and to a need for intensive care [65].

Recently hyperpolarised helium magnetic resonance imaging (^3^He MRI) has been used to assess the structure and the ventilation in distal airways using both static and dynamic protocols [66]. Even if there is some interesting evidence also in asthmatic patients [67,68], this technique is difficult to perform in the routine clinical evaluation.

Some difficulties exist also for nuclear medicine tests, such as two-dimensional (2-D) gamma scintigraphy, single photon emission computed tomography (SPECT) and positron emission tomography (PET). They showed interesting evidence in evaluating the ventilation and the distribution of the inhaled drugs [69,70,71,72,73,74], but the cost/effectiveness ratio is still disadvantageous in the patient’s follow up.

## 4. Treatment: How Could We Reach the Peripheral Airways?

The awareness of the small airways’ involvement in the pathogenesis of asthma is a cornerstone for the management of this disease [75], with a particular importance in the not well-controlled patients. A review from Usmani et al. [76] described an overall prevalence of small airways disease in adult asthma, of 50–60% across all asthma severities and even in milder disease. The presence of small airways disease across all severities of asthma was also confirmed by Postma et al. in a recent prospective cohort study [77].

Treatment with inhaled corticosteroids alone or in combination with LABA is at the basis of asthma management [1] but, despite the high dosages of ICS, a high number of patients does not reach optimal disease control. The success of a therapy depends on several factors as described before, but the amount of drug reaching the peripheral lungs plays a substantial role in asthma patients. Therefore, the best therapeutic choice is the one that allows one to reach both proximal and distal airways. The ability of the inhaled drugs to reach the periphery is strictly linked to the size of the molecules: the thinner they are, the easier they can reach small airways [78]. After the outlawing of chlorofluorocarbons (CFC), different studies focused on the development of new propellants, drug formulations and inhaler devices. The hydrofluoroalkanes (HFA) became promising alternative propellant molecules. Aerosol formulations containing HFA have shown a greater peripheral lung deposition than those containing CFC [79,80].

In the recent decades, formulations containing particles with a diameter smaller than 1.5 µm have been developed [81,82] and, in particular, an HFA-propelled extrafine fixed combination of beclometasone dipropionate/formoterol (BDP/F) delivered via pressurized metered dose inhaler (pMDI) was introduced in the drug market [83]. This extrafine solution enables drug particles to reach both central and peripheral airways, optimizing treatment of the inflammatory process with an increase of peripheral lung deposition with respect to the initial dose, resulting in reduced systemic exposure [84]. Furthermore, pMDI does not require a deep inspiration for an effective distribution of the drug in the airways due to the presence of the HFA propellant. A deep inhalation is instead essential with the use of dry powder inhalers. It is usual in clinical practice not to recommend dry powder inhalers (DPIs) to patients unable to perform an effective inhalation [85].

Several studies were performed to assess the efficacy of extrafine formulations compared to non-extrafine, starting from inhaled corticosteroids. These studies revealed that beclometasone delivered in extrafine particles improves asthma control in terms of non-recorded hospital attendance for asthma, use of oral corticosteroids or antibiotics, producing real life benefits [86,87]. Other studies showed that extrafine ICS were more effective than non-extrafine in the regulation of functional and inflammatory parameters that reflect small airways impairment especially in improving acinar ventilation heterogeneity [88] and in reducing regional air trapping [89].

Clinical trials were conducted using the extrafine fixed combination of BDP/F delivered via HFA pMDI. In two separate studies performed on more than 200 patients, it was observed that this combination was an effective and safe alternative for the treatment of asthma compared to non-extrafine combinations of budesonide/formoterol (BUD/F) and fluticasone/salmeterol (FP/S) in terms of lung function improvement as measured by morning peak expiratory flow, symptom score and rescue medication use [57,90]. In a real-life study conducted on 111 patients [91], Muller et al. showed that pMDI extrafine BDP/F combination was more effective in improving asthma control compared to DPIs formulated with larger particles. Moreover, in a randomized clinical trial involving 645 patients with moderate to severe not controlled asthma, Huchon et al. showed that BDP/F in a single inhaler was significantly superior to separate components in improving asthma control in terms of symptoms, and to beclomethasone dipropionate alone in improving both asthma control and lung function measured as morning peak expiratory flow [92]. To check if pMDI extrafine BDP/F was effective on small airways impairment, Scichilone et al. found a trend toward improvement in closing capacity assessed by single breath nitrogen test, in a randomized trial involving 30 asthmatic patients treated for 12 weeks with either extra-fine BDP/F 400/24 µg daily or fluticasone propionate/salmeterol (FP/S) 500/100 µg daily [93]. In a real-life clinical study, 59 patients switched from fluticasone/salmeterol and budesonide/formoterol DPI to extrafine BDP/F. After 8 weeks of treatment, the subjects with highest rise in FVC% showed also a significant improvement in inflammation evaluated as exhaled breath temperature, blood eosinophils, and C-reactive protein [94]. Bulac et al. evaluated the effect of extrafine BDP/F in asthmatic patients on exhaled NO and on S_acin_ and S_cond_ values, indices of small airways function, in comparison with budesonide/formoterol and fluticasone/salmeterol DPI. They found a significant decrease in eNO levels and a significant improvement in S_acin_ values in asthma patients treated with BDP/F-HFA [95].

As regard to long-acting muscarinic antagonists (LAMA), they are effective for people with COPD and are also suggested in asthma in addition to LABA/ICS [1]. In particular, tiotropium has been shown to have additional benefits in reducing the need for rescue oral steroids in people with severe asthma [96]. However, there are no clinical trials showing the effectiveness of LAMA in the improvement of small airways impairment in asthmatic subjects.

Speaking of parental therapy, we focus our interest on the role of oral anti-inflammatory anti-leukotriene agents. The fact that the leukotriene receptors are expressed at higher levels in small airways fibroblasts can explain a stronger effect of one of them, montelukast, in the deeper lung [97]. Kraft et al. studied asthmatic patients with air trapping (RV >140% of predicted value) and observed a significant improvement in symptoms of wheezing, dyspnea, and cough after treatment with montelukast [98]. In the same year, Zeidler et al., using a HRCT at residual volume before and after a challenge test with methacholine, assessed a month effect of montelukast treatment in 16 mild to moderate steroid-naive patients. They showed, compared to the placebo group, a reduction in air trapping in the pre-methacholine images and a symptoms improvement even if there were no changes in conventional functional parameters [99]. In contrast, the study of Gelb and colleagues, that evaluated the addition of anti-leukotriene therapy (zileuton 2400 mg daily) for 4 weeks in 19 stable moderate-severe patients with asthma in treatment with ICS/LABA (FP/Salm 500/100 μg daily) for at least 1 year, did not show a significant effect on either distal/alveolar or central/bronchial airway nitric oxide concentrations [100].

Recently, the effect of biologic drugs on small airways has been studied as well. In particular, Farah et al. showed an improvement in ventilation inhomogeneity measured with multiple breath nitrogen washout, in twenty subjects with severe eosinophilic asthma treated with mepolizumab. Moreover, they found that the improvement in small airways function correlates with the improvement in symptom score [101].

## 5. Conclusions

It is now widely established that the inflammation affects both proximal and peripheral lung in asthmatic patients with a real impact on the clinical and therapeutic aspects. Different inflammatory, functional, and radiological parameters have been tested to describe the distal airways impairment in asthmatic subjects. In particular, the appraisal of inflammation by alveolar nitric oxide assessment and the adoption of new imaging techniques for the evaluation of air trapping and thickening of small airways wall, have proved to be useful and suitable in clinical practice. Future studies should be directed to understand if the early detection of small airways abnormalities could be helpful as a prognostic factor for a more severe disease. Anyway, since the contribution of the distal lung compartment to the pathophysiology of asthma is certain, the asthmatic patients, in particular those difficult to treat, should be subjected to an assessment of peripheral airways function and inflammation, and should be treated with drugs able to reach the most peripheral lung.

## Figures and Tables

**Table 1:** Methods of assessment of small airways impairment.

Methods	Small Airway Measures	Advantages	Disadvantages
Spirometry	FVC/SVC, FEV3, FEV6, FEF25-75	Non-invasive; Easy to perform; Widely available	Highly variability
Body plethysmography	RV/TLC, DLCO, Raw	Non-invasive; Easy to perform	Not much evidence about it; Not widely available
Impulse oscillometry	R5–R20, X5, AX, Fres	Non-invasive; Easy to perform	Not widely available
Single breath nitrogen washout and Multiple breath washout test	Slope phase III, CV, CC, Sacin, Scond	Non-invasive; Good sensitivity and reproducibility	Not widely available
Imaging	Air trapping, airway wall thickness; Regional ventilation defects; Non ventilated lung volume	Non-invasive	Exposure to radiations; Costly
Exhaled nitric oxide at multiple exhalation flows	Alveolar NO	Non-invasive; Good reproducibility	Not direct assessment; Requires computational extrapolation
Sputum induction	Cellular population, inflammatory markers	Non-invasive; Direct assessment	Low reproducibility
Transbronchial biopsy	Cellular population	Direct assessment	Invasive
Bronchoalveolar lavage	Cellular population	Direct assessment	Invasive
